# Linear Programming and Fuzzy Optimization to Substantiate Investment Decisions in Tangible Assets

**DOI:** 10.3390/e22010121

**Published:** 2020-01-19

**Authors:** Marcel-Ioan Boloș, Ioana-Alexandra Bradea, Camelia Delcea

**Affiliations:** 1 Department of Finance and Banks, University of Oradea, 410087 Oradea, Romania; marcel.bolos@softscape.ro; 2 Department of Informatics and Cybernetics, Bucharest University of Economic Studies, 010374 Bucharest, Romania; ioana.bradea@softscape.ro

**Keywords:** tangible assets, investment decisions, situation analysis, graphical method, primal simplex algorithm, fuzzy coefficients and decision variables, fuzzy triangular numbers

## Abstract

This paper studies the problem of tangible assets acquisition within the company by proposing a new hybrid model that uses linear programming and fuzzy numbers. Regarding linear programming, two methods were implemented in the model, namely: the graphical method and the primal simplex algorithm. This hybrid model is proposed for solving investment decision problems, based on decision variables, objective function coefficients, and a matrix of constraints, all of them presented in the form of triangular fuzzy numbers. Solving the primal simplex algorithm using fuzzy numbers and coefficients, allowed the results of the linear programming problem to also be in the form of fuzzy variables. The fuzzy variables compared to the crisp variables allow the determination of optimal intervals for which the objective function has values depending on the fuzzy variables. The major advantage of this model is that the results are presented as value ranges that intervene in the decision-making process. Thus, the company’s decision makers can select any of the result values as they satisfy two basic requirements namely: minimizing/maximizing the objective function and satisfying the basic requirements regarding the constraints resulting from the company’s activity. The paper is accompanied by a practical example.

## 1. Introduction

In the current economic context, companies will have to solve a rather complex decision problem, respectively, to consider capital assets in tangible assets that ensure the best combination between the economic performance of the tangible assets, the economic benefits generated by the tangible assets during the useful economic life and to take into account the constraints that the company has due to the limited nature of the resources. Solving this complex decision problem is the main purpose and motivation for this research paper.

The major contribution of the paper consists in solving a complex problem within the company, namely, to obtain a favorable relationship between the economic benefits of the tangible assets, the acquisition cost, and the constraints of the company. This complex problem was solved with a hybrid model which uses two modern tools:

- linear programming, through which it is ensured that conditions are met for the tangible assets, namely: the fulfillment of the objective function condition that can be to minimize the cost of acquiring the tangible assets or to maximize the economic benefits generated by them; and at the same time the fulfillment of the restrictions that refer to the limited character of the resources. For example, limited investment budget, limited budget for maintenance expenses or limited areas destined to the production activities, etc.,
- the fuzzy optimization necessary for modeling the technical and economic criteria that are specific to the tangible assets. The fuzzy modeling for the acquisition criteria specific to the tangible assets has a number of advantages, such as ensuring the comparability between the acquisition criteria of the assets with different units of measure, ensuring the hierarchy of the tangible assets according to their economic performance, ensuring the value ranges stratification for the analysis of the acquisition criteria, etc.

The novelty of the approach consists of the introduction of fuzzy variables and fuzzy coefficients both in the objective function and in the problem constraints, in order to ensure the decision-making framework of the company’s investments in tangible assets. This is possible since the triangular fuzzy numbers, that were the basis of the fuzzy modeling for the implementation of the graphical method and the primal simplex algorithm, are presented as value ranges. Any value in this range, which is the solution of the linear programming problem satisfies both the requirements resulting from the objective function and the constraints of the problem. Moreover, the use of triangular fuzzy numbers allows the characterization of the objective function coefficients, the decision variables or the constraint variables of the problem, with the help of linguistic variables as well as the precise measurement of their vague character.

Linear programming and fuzzy optimization as a solution to the problem identified above, finally lead to the substantiation of the investment decision in tangible assets that allows the immobilization of capital for different periods of time so that the economic benefits obtained from the use of the asset in its organic activity is maximum, also respecting the constraints.

The innovative nature of this research is represented precisely by the introduction of fuzzy coefficients and fuzzy variables in the decision-making process of companies’ investments with implementation of the graphical method and the primal simplex algorithm.

The paper is structured in seven sections. The first one is dedicated to the introduction part of the research, emphasizing the motivation, the advantages, and the novelty character of the paper. The Section 2 presents the state-of-the-art in the domain of linear programing and fuzzy programming, by analyzing the ISI Web of Science database. The Section 3 is dedicated to fuzzy modeling and its main advantages for solving investment decisions.

Within the Section 4, the graphical method for solving linear programming problem with fuzzy optimization is presented, while in the Section 5 the primal simplex algorithm with fuzzy variables is proposed for solving minimization problems. In the Section 6, the hybrid model is tested, and the main conclusions of the research are stipulated in Section 7. The paper ends with the references list.

## 2. State-of-the-Art

Over the time, the development and advancements made in linear programing have produced a series of advantages for companies acting in different parts of the world. Even though this approach comes with a series of assumptions, such as the linearity of the mathematical functions with the model, it has been extensively used in practical applications as it provides the best (optimal) solution when considering the limited amount of resources and by taking into account all the feasible alternatives.

Since 1951 when the first programmed solution code based on Dantzig’s simplex method was developed, a series of papers have discussed the different aspects related to linear programming and simplex method such as: computational aspects [1,2], adapting the simplex approach to bilevel linear programming [3,4], applying simplex method for singularly perturbed linear programs [5], determining the conditions for reachability on a simplex algorithm [6], and computing experimental designs using simplex method [7].

As for the applications, a series of papers envisioned the fusion between artificial intelligence techniques and simplex algorithm for solving the issues under investigation. Among the artificial intelligent techniques used alongside the simplex algorithm are genetic algorithms [8,9,10,11,12], support vector machines [13,14], neural networks [15,16], and fuzzy sets theory [17].

Nevertheless, the scientific community has extensively used artificial intelligence techniques in decision-making problems as these techniques allow a proper data analysis through specific intelligent algorithms [18]. Among them, fuzzy theory has a particular place as it succeeds in ensuring the comparability of the variables and the used criteria, providing often robust algorithms and a simple reasoning process [19]. Even more, in some cases, a series of randomized optimization algorithms can be considered for the fuzzy logic design as suggested in [20].

A series of fuzzy numbers have been proposed and used over the time in economic analysis [21], such as triangular fuzzy numbers [22,23], interval fuzzy numbers [24], type-2 fuzzy numbers [25,26], and trapezoidal fuzzy numbers [27]. Also, with the appearance of the neutrosophic theory [28,29,30], neutrosophic fuzzy numbers were created and used in economic applications related to the decision-making process [23,31].

According to Wang and Tong [21], the triangular fuzzy numbers are the most widely used in decision-making problems as their membership functions are better modeled for mapping different uncertainty levels. In their work, the authors focused on the multiplicative consistency and group decision-making when the triangular fuzzy numbers were considered and proposed a procedure for solving group decision-making problems.

In the same area of decision-making, Karimi et al. [32] proposed a method involving a linear mathematical model, fuzzy triangular numbers, and considering best-worst situations. As a result, the authors underlined a high efficiency of the proposed method when compared with a fuzzy analytic hierarchy process (AHP).

Liu et al. [33] focused their research on the construction of the triangular fuzzy additive reciprocal matrixes used in a group decision model. The authors proposed a new algorithm for triangular fuzzy additive reciprocal matrices and used a numerical example in order to support their proposed approach.

Wan et al. [34] proposed new generalized aggregation operators for triangular intuitionistic fuzzy numbers and presented some applications for multi-group decision-making. Other papers in the area of decision-making which considers the use of triangular fuzzy numbers are, but not restricted to: Krohling and Pacheco [35] by providing an improved accuracy function; Wan et al. [36] by extending the Vlsekriterijumska Optimizacija I Kompromisno Resenje (VIKOR) method; Yuan and Luo [37] by developing a novel intuitionistic fuzzy entropy and evidential reasoning; Chen and Li [38] by considering a dynamic model; and Qin et al. [39] by the extending TODIM (acronym in Portuguese for interactive and multicriteria decision-making) method.

Referring strictly to the fuzzy decision variables used within the classical operations research problems, it can be observed that a series of researchers have chosen the fuzzy representation of decision variables and tried to solve real-life applications by combining the classical operations research theory with the advantages offered by the fuzzy approach. Transportation problems are a major category of problems within the operations research area which have been extensively studied using the fuzzy numbers approach. As mentioned by Ebrahimnejad [40] in “real-life situations, the parameters of transportation problems may not be known precisely because of uncontrollable factors”, which makes the use of the fuzzy numbers, for representing transportation costs, supply, and demand quantities, more appealing. After defining the new fuzzy transportation problem, the author converted the proposed problem into four transportation problems, which were afterwards solved through the use of a standard simplex algorithm [40]. As a result, the author stated that proceeding in this way, the efficiency of solving this category of problems increased and was kept as simple as possible.

Also, in the area of transportation problems, Baykasoglu and Subulan [41] proposed a fuzzy arithmetic approach for solving fully fuzzy balanced and unbalanced transportations problems when all the parameters and decision variables are fuzzy numbers. Based on the decision maker’s risk attitude, various fuzzy solutions can be generated. By considering a series of practical applications presented in their paper, the authors underlined the fact that more precise solutions to transportation problems are received when the decision maker is either risk-averse or partially risk-averse, while in the case of risk-seekers, the proposed approach provided acceptable solutions [41].

Kocken and Sivri [42] considered in their research a transportation problem in which all the cost coefficients, supplies, demands, and conveyances are assumed to be fuzzy quantities represented through triangular fuzzy numbers and proposed a method for determining all the optimal solutions. Based on a numerical example, the authors demonstrated that the proposed procedure produces good results and provides a more general perspective over the decision-making process.

More generally, referring to the whole class of linear programming problems, Ebrahimnejad and Tavana [43] stated that, over time, “fuzzy sets theory has been extensively used to represent imprecise data in linear programming by formalizing the inaccuracies inherent in human decision-making”. In this context, the authors proposed a general approach to solve fuzzy linear programming problems when the coefficients of the objective function and the values of the B vector are symmetric trapezoidal fuzzy numbers. Even in this case, the authors stated that the proposed method is simpler and more efficient from a computational point of view than other approaches from the literature.

A special class of fuzzy linear programming problems, consisting of both fuzzy inequalities and fuzzy coefficients and decision variables, described through trapezoidal fuzzy numbers, was considered by Stanojevic et al. [44]. The proposed method was based on an order relation used for ranking the intervals of the optimization problem. Using a numerical example, the authors showed that for a given fixed value of the acceptance degree established by the decision maker, the decision maker can make more informed decisions in relationship with the problem needed to be completed [44].

Other results in the area of sensitivity analysis in fuzzy number linear programming were obtained in a paper written by Ebrahimnejad [45]. The author used sensitivity analysis for determining changes in the optimal solution in which changes in data may occur [45].

Based on the works mentioned above in the area of fuzzy linear programming, it can be observed that a fuzzy approach to linear programming problems can be beneficial to the decision maker as this approach provides insight into the analyzed problem while also modeling the problem more closely to the considered real-life situation.

## 3. Fuzzy Modeling and the Company’s Investment Decision

The investment decisions in tangible assets represent capital assets for different periods of time in order to be used in the organic activity of the company, but also to obtain economic benefits that consolidate the financial status of the company. A modern view on the tangible assets acquisition policy would involve capital assets that enable the stakeholders to recover the invested capital from the economic benefits that these categories of assets produce during their use. Therefore, the concept of investment in assets is added to the concept of economic benefits generated by any category of tangible assets that were the subject of a purchase on the market.

Thus, the corporal assets of the companies are based on two fundamental concepts, namely: the concept of economic performance that is the result of the economic and technical characteristics that a corporal asset has, and the concept of economic benefits which in turn is the result of the cash-flows generated by a tangible asset during its use in the organic activity of the company.

The economic benefits of a tangible asset are influenced by its economic performance. Therefore, a tangible asset with poor economic performance and an acceptable purchase price will generate low economic benefits that may cause problems for the financial sustainability of the asset.

The economic performance of a tangible asset is the result of the economic characteristics which can include the acquisition cost of the asset, its production capacity, the productivity of the tangible asset, as well as the technical characteristics which may also include maintenance expenses, operating expenses of the asset, the useful life of the asset, or the warranty duration provided by supplier.

In this respect, a company which decides to invest in tangible assets, by acquiring them from a competitive market, has to ensure the best combination between the economic characteristics and technical characteristics of the asset, in order to maximize the benefits generated by the asset during its lifetime. Following this rule, the company can achieve a reasonable ratio between the assets’ acquisition costs and the economic benefits generated, without affecting the financial sustainability of the tangible asset, but also contributing to the consolidation of the financing status of the company.

The economic benefits and the economic performance generated by the tangible assets consider the constraints which emerge from the organic activity of the company. These constraints mainly refer to the resources available to the company. No matter the type of resource—financial, material, human, or technical—they are limited in nature.

In this context, the fuzzy variables and fuzzy coefficients are used in the application of the primal simplex algorithm, which allows the company to sort the tangible assets according to the criteria hierarchy for the acquisition of tangible assets, ensuring the comparability of the criteria for the acquisition of the tangible assets with different units of measurement and allowing the flexibility of the decision-making process resulting from the acquisition of tangible assets on a competitive market. In the following, the fuzzy numbers are discussed along with their use for constraints and acquisition criteria.

### 3.1. Fuzzy Numbers for Acquisition Criteria and Constraints

Fuzzy modeling, in substantiating the investment decisions of companies, intervenes, as mentioned above, in shaping the acquisition criteria of tangible assets but also in modeling the company specific restrictions. Fuzzy modeling of both the acquisition criteria and constraints has several advantages for substantiating investment decisions at the level of the companies.

For fuzzy modeling, three categories of fuzzy numbers are used, namely: fuzzy numbers for modeling economic asset acquisition criteria ($C_{e}$), fuzzy numbers for modeling technical asset acquisition criteria ($C_{t}$), as well as fuzzy numbers for modeling company constraints, or restrictions ($C_{R}$). The fuzzy numbers used are in the form of triangular fuzzy numbers as they best describe the acquisition criteria for tangible assets, or company constraints.

Definition 1: Let the discourse universe be represented by the multitude of economic criteria for the acquisition of assets ($C_{e}$) that can take various forms (acquisition cost, production capacity, productivity), and F [0,1] the set of rules for fuzzy numbers. The fuzzy number ${〖(C〗}_{e})$ is called the triangular fuzzy number of the economic criteria for the asset acquisition and takes the form: $C_{e}=\left\{c_{e},\frac{\mu_{c_{e}}}{c_{e}}\in C_{e}\right\},$ where $\mu_{c_{e}}:C_{e}\to \left[0,1\right]$ if the membership function is defined by the relation [23,46,47]:

(1) $$\mu_{c_{e_{\left(e\right)}}}=\left\{ \begin{array}{c} 1-\frac{c_{e_{x}}-c_{e_{a}}}{c_{e_{b}}-c_{e_{a}}},\;for\;c_{e_{a}}\leq c_{e_{x}}\leq c_{e_{b}}\\ 1,\;for\;c_{e_{x}}=c_{e_{b}}\\ 1-\frac{c_{e_{c}}-c_{e_{x}}}{c_{e_{c}}-c_{e_{b}}},\;for\;c_{e_{b}}\leq c_{e_{x}}\leq c_{e_{c}}\\ 0,\;for\;values\;outside\;the\;range\;[c_{e_{c}};c_{e_{a}}] \end{array}\right.$$

Definition 2: Let the triangular fuzzy number that defines the set of economic criteria for the acquisition of tangible assets be in the following form: $C_{ei}=\left\{c_{ei},\frac{\mu_{c_{ei}}}{c_{ei}}\in C_{ei}\right\}$ for any $i=\bar{1,n}$. We will say that the set ${\left[C_{e}\right]}^{\alpha }=\left[C_{e_{1}}\left(\alpha \right);C_{e_{2}}\left(\alpha \right)\right]$ for any *α* ∈ [0,1] is called the level set of the triangular fuzzy number [46,48] $C_{e},$ where:

(2) $$C_{e_{1}}\left(\alpha \right)=\left(c_{e_{b}}-c_{e_{a}}\right)\alpha +c_{e_{a}}$$

(3) $$C_{e_{2}}\left(\alpha \right)=c_{e_{c}}-\left(c_{e_{c}}-c_{e_{b}}\right)\alpha$$

Observation 1: The economic criteria can take values in the range: $\left[c_{e_{x}}-c_{e}\leq c_{e}\leq c_{e}+c_{e_{a}}\right]$, where $c_{e_{x}}\in R$.

Definition 3: Let the discourse universe consist of the technical criteria set for asset acquisition ($C_{t}$) and the set of rules F [0,1] valid for fuzzy numbers. The fuzzy number $C_{t}$ is called the triangular fuzzy number of the form $C_{t}=\left\{c_{t},\frac{\mu_{c_{t}}}{c_{t}}\in C_{t}\right\},$ where $\mu_{c_{t}}:C_{t}\to \left[0,1\right],$ if the membership function is defined by the relation [23,46,47]:

(4) $$\mu_{c_{t_{\left(x\right)}}}=\left\{ \begin{array}{c} 1-\frac{c_{t_{x}}-c_{t_{a}}}{c_{e_{b}}-c_{e_{a}}},\;for\;c_{t_{a}}\leq c_{t_{x}}\leq c_{t_{b}}\\ 1,\;for\;c_{t_{x}}=c_{t_{b}}\\ 1-\frac{c_{t_{c}}-c_{t_{x}}}{c_{t_{c}}-c_{t_{b}}},\;for\;c_{t_{b}}\leq c_{t_{x}}\leq c_{t_{c}}\\ 0,\;for\;values\;outside\;the\;range\;[c_{t_{c}};c_{t_{a}}] \end{array}\right.$$

Observation 2: The technical criteria for asset selection can take various forms such as: maintenance expenses ($E_{mi}$), operating expenses of the asset ($E_{oi}$), the useful life of the asset ($U_{li}$), or the warranty duration provided by the supplier ($W_{di}$).

Definition 4: It is considered the level set of the fuzzy triangular number $C_{ti}=\left\{c_{ti},\frac{\mu_{c_{ti}}}{c_{ti}}\in C_{ti}\right\},$ for any $i=\bar{1,n}$, of the form: ${\left[C_{t}\right]}^{\alpha }=\left[C_{t_{1}}\left(\alpha \right);C_{t_{2}}\left(\alpha \right)\right]$ for α∈[0,1] where:

(5) $$C_{t_{1}}\left(\alpha \right)=\left(c_{t_{b}}-c_{t_{a}}\right)\alpha +c_{t_{a}}$$

(6) $$C_{t_{2}}\left(\alpha \right)=c_{t_{c}}-\left(c_{t_{c}}-c_{t_{b}}\right)\alpha$$

Observation 3: The technical criteria can take values in the range $\left[c_{t_{x}}-c_{t}\leq c_{t}\leq c_{t}+c_{t_{x}}\right],$ where $c_{t_{x}}\in R$.

Definition 5: Let the discourse universe consist of the constraints set determined by the resource limited character ($C_{R}$) and the set of rules F [0,1] valid for fuzzy numbers. The fuzzy number $C_{R}$ is called the triangular fuzzy number of the form $C_{R}=\left\{c_{R},\frac{\mu_{c_{R}}}{c_{R}}\in C_{R}\right\}$ where $\mu_{c_{R}}:C_{R}\to \left[0,1\right]$, if the membership function is defined by the relation [23,46,47]:

(7) $$\mu_{c_{R_{\left(x\right)}}}=\left\{ \begin{array}{c} 1-\frac{c_{R_{x}}-c_{R_{a}}}{c_{R_{b}}-c_{R_{a}}},\;for\;c_{R_{a}}\leq c_{R_{x}}\leq c_{R_{b}}\\ 1,\;for\;c_{R_{x}}=c_{R_{b}}\\ 1-\frac{c_{R_{c}}-c_{R_{x}}}{c_{R_{c}}-c_{R_{b}}},\;for\;c_{R_{b}}\leq c_{R_{x}}\leq c_{R_{c}}\\ 0,\;for\;values\;outside\;the\;range\;[c_{t_{c}};c_{t_{a}}] \end{array}\right.$$

Definition 6: It is considered the level set of the triangular fuzzy number [23,46,47,49], $C_{R}=\left\{c_{R},\frac{\mu_{c_{R}}}{c_{R}}\in C_{R}\right\},$ for any $i=\bar{1,n}$ of the form: ${\left[C_{R}\right]}^{\alpha }=\left[C_{R_{1}}\left(\alpha \right);C_{R_{2}}\left(\alpha \right)\right]$ for any α∈[0,1], where:

(8) $$C_{R_{1}}\left(\alpha \right)=\left(c_{R_{b}}-c_{R_{a}}\right)\alpha +c_{R_{a}}$$

(9) $$C_{R_{2}}\left(\alpha \right)=c_{R_{c}}-\left(c_{R_{c}}-c_{R_{b}}\right)\alpha$$

Observation 4: The constraints are determined by the limited character of financial resources ($C_{Rf}$), by the limited character of material or technical resources (C_Rt), but also by the limited character of human resources ($C_{RU}$).

### 3.2. Common Rules for Fuzzy Modeling

The rules for fuzzy modeling were introduced due to the diversity of criteria that intervenes in substantiating investment decisions at the company level, but also to facilitate the calculations involved in fuzzy modeling. These criteria, as mentioned above, include the economic criteria for the assets acquisition, the technical criteria for the assets acquisition, and the constraints determined by the organic activity of the company [46,47,49].

**Figure 1:**
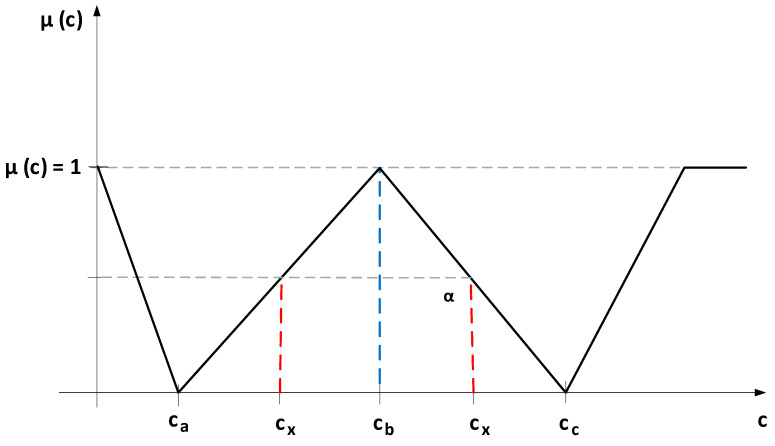
The triangular fuzzy number *C* used in fuzzy modeling.

Rule 1: The fuzzy triangular numbers are represented by:

(a) the economic criterion for the assets acquisition $C_{e}=\left\{c_{e},\frac{\mu_{c_{e}}}{c_{e}}\in C_{e}\right\}$, where $\mu_{c_{e}}:C_{e}\to \left[0,1\right]$;
(b) the technical criterion for the assets acquisition $C_{t}=\left\{c_{t},\frac{\mu_{c_{t}}}{c_{t}}\in C_{t}\right\}$, where $\mu_{c_{t}}:C_{t}\to \left[0,1\right]$;
(c) the limited character of the resources available to the company $C_{R}=\left\{c_{R},\frac{\mu_{c_{R}}}{c_{R}}\in C_{R}\right\}$, where $\mu_{c_{R}}:C_{R}\to \left[0,1\right]$;

and noted in the linear programming calculations with $=\left\{c,\frac{\mu_{c}}{c}\in C\right\},$ where $\mu_{c}:C\to \left[0,1\right],$ representing the triangular fuzzy number for the linear programming problems constraints (according to Figure 1), for which the membership function is [23,47]:

(10) $$\mu_{\left(c\right)}=\left\{ \begin{array}{c} 1-\frac{c_{x}-c_{a}}{c_{b}-c_{a}},\;for\;c_{a}\leq c_{x}\leq c_{b}\\ 1,\;for\;c_{x}=c_{b}\\ 1-\frac{c_{c}-c_{x}}{c_{c}-c_{b}},\;for\;c_{b}\leq c_{x}\leq c_{c}\\ 0,\;for\;values\;outside\;the\;range\;[c_{c};c_{a}] \end{array}\right.$$

Observation 5: In situations where linear programming restrictions require the use of specific criteria for tangible assets acquisition or constraints, these are represented by the three triangular fuzzy numbers previously presented [46,50], of the form: $C_{e}=\left\{c_{e},\frac{\mu_{c_{e}}}{c_{e}}\in C_{e}\right\}$, $C_{t}=\left\{c_{t},\frac{\mu_{c_{t}}}{c_{t}}\in C_{t}\right\},$ and $C_{R}=\left\{c_{R},\frac{\mu_{c_{R}}}{c_{R}}\in C_{R}\right\}$.

Rule 2: The level set for the triangular fuzzy number $C=\left\{c,\frac{\mu_{c}}{c}\in C\right\}$, where $\mu_{c}:C\to \left[0,1\right]$ is of the form: ${\left[C\right]}^{\alpha }=\left[C_{1}\left(\alpha \right);C_{2}\left(\alpha \right)\right],$ for any *α* ∈ [0,1] where:

(11) $$C_{1}\left(\alpha \right)=\left(c_{b}-c_{a}\right)\alpha +c_{a}$$

(12) $$C_{2}\left(\alpha \right)=c_{c}-\left(c_{c}-c_{b}\right)\alpha$$

Rule 3: Let two fuzzy numbers be of the form: $C_{1}=\left(c_{a1},c_{b1}\right)$ and $C_{2}=\left(c_{a2},c_{b2}\right)$ with $a_{1},a_{2},b_{1},b_{2}\in R$. The following arithmetic operations are valid [46,47,51]:

- Fuzzy numbers addition
  (13) $$C_{1}+C_{2}=\left[c_{a1},c_{b1}\right]+\left[c_{a2},c_{b2}\right]=\left[c_{a1}+c_{a2},c_{b1}+c_{b2}\right]$$
- Fuzzy numbers subtraction
  (14) C_1−C_2=[c_a1,c_b1]−[c_a2,c_b2]=[c_a1−c_a2,c_b1−c_b2]
- Fuzzy numbers multiplication
  (15) $$C_{1}\times C_{2}=\left[c_{a1},c_{b1}\right]\times \left[c_{a2},c_{b2}\right]=\;\left[\min\left(c_{a1}c_{a2},\;c_{a1}c_{b2},c_{b1}c_{a2},c_{b1}c_{b2}\;\right),\max\left(c_{a1}c_{a2},\;c_{a1}c_{b2},c_{b1}c_{a2},c_{b1}c_{b2}\right)\right]$$
- Fuzzy numbers division
  (16) $$\frac{C_{1}}{C_{2}}=\frac{\left[c_{a1},c_{b1}\right]}{\left[c_{a2},c_{b2}\right]}=\left[\min\left(\frac{c_{a1}}{c_{a2}},\frac{c_{a1}}{c_{b2}},\frac{c_{b1}}{c_{a2}},\frac{c_{b1}}{c_{b2}}\right),\max\left(\frac{c_{a1}}{c_{a2}},\frac{c_{a1}}{c_{b2}},\frac{c_{b1}}{c_{a2}},\frac{c_{b1}}{c_{b2}}\right)\right]$$
- The inverse of fuzzy numbers
  (17) $$\frac{1}{C_{1}}=\frac{1}{c_{a1},c_{b1}}=\left[\min\left(\frac{1}{c_{a1}};\frac{1}{c_{b1}}\right),\max\left(\frac{1}{c_{a1}};\frac{1}{c_{b1}}\right)\right]$$
  (18) $$\frac{1}{C_{2}}=\frac{1}{c_{a2},c_{b2}}=\left[\min\left(\frac{1}{c_{a2}};\frac{1}{c_{b2}}\right),\max\left(\frac{1}{c_{a2}};\frac{1}{c_{b2}}\right)\right]$$

Rule 4: The level set of the triangular fuzzy number $C=\left\{c,\frac{\mu_{c}}{c}\in C\right\}$ for any $i=\bar{1,n}$ is of the form: ${\left[C\right]}^{\alpha }=\left[C_{1}\left(\alpha \right);C_{2}\left(\alpha \right)\right]$ [47], for any *α* ∈ [0,1] where:

(19) $$C_{1}\left(\alpha \right)=\left(c_{b}-c_{a}\right)\alpha +c_{a}$$

(20) $$C_{2}\left(\alpha \right)=c_{c}-\left(c_{c}-c_{b}\right)\alpha$$

Rule 5: The average value of the fuzzy number $C=\left\{c,\frac{\mu_{c}}{c}\in C\right\}$, where $\mu_{c}:C\to \left[0,1\right]$ is of the form [46,47,51]:

(21) $$E_{f}\left(C_{i}\right)=\frac{1}{2}\int_{0}^{1}\left(C_{1}\left(\alpha \right)+C_{1}\left(\alpha \right)\right)f\left(\alpha \right)d\alpha$$

where *f* (*α*) is a weight function *f*: [0,1] → R which satisfies the following conditions:

- It is a monotonically increasing function, respectively ∀ x,y∈R and x≤y, it follows that f(x)≤f(y);
- Checks the normality condition, namely: $\int_{0}^{1}f\left(\alpha \right)d\alpha =\int_{0}^{1}2\alpha d\alpha =2\frac{\alpha^{2}}{2}{│}_{0}^{1}=1..$

The weigh function is used to calculate the main indicators of the fuzzy numbers, respectively the arithmetic mean, the squared deviations from the mean, and the covariance. The most commonly used weight function is *f* (*α*) = 2*α*, which meets the conditions imposed above, namely:

- It is a monotonically increasing function. $\forall \;\alpha_{1},\alpha_{2}\in R$ with $\alpha_{1}\leq \alpha_{2}$ results that $f\left(\alpha_{1}\right)\leq f\left(\alpha_{2}\right)$. From this condition it appears that $2\alpha_{1}\leq 2\alpha_{2}$, respectively $\alpha_{1}\leq \alpha_{2}$.
- Checks the normality condition, namely: $\int_{0}^{1}f\left(\alpha \right)d\alpha =\int_{0}^{1}2\alpha d\alpha =2\frac{\alpha^{2}}{2}{│}_{0}^{1}=1$.

Rule 6: The average value of the fuzzy triangular number of the form $C=\left\{c,\frac{\mu_{c}}{c}\in C\right\},$ is given by the relation [46,47,51]:

(22) $$E_{f}\left(C_{i}\right)=\frac{1}{6}\left(c_{a1}+c_{c1}\right)+\frac{2}{3}c_{b1}$$

Demonstration: According to rule 6, the average value of the fuzzy number *C* is calculated with the relation:

$$E_{f}\left(C_{i}\right)=\frac{1}{2}\int_{0}^{1}\left(C_{1}\left(\alpha \right)+C_{1}\left(\alpha \right)\right)f\left(\alpha \right)d\alpha$$

It is determined:

$$C_{1}\left(\alpha \right)+C_{2}\left(\alpha \right)=\left(c_{b1}-c_{a1}\right)\alpha +c_{a1}+c_{c1}-\left(c_{c1}-c_{b1}\right)\;=c_{b1}\alpha -c_{a1}\alpha +c_{a1}+c_{c1}-c_{c1}\alpha +c_{b1}\alpha \;C_{1}\left(\alpha \right)+C_{2}\left(\alpha \right)=c_{a1}\left(1-\alpha \right)+2c_{b1}\alpha +c_{c1}\left(1-\alpha \right)$$

The above formula can be written as:

$$C_{1}\left(\alpha \right)+C_{2}\left(\alpha \right)=\left(1-\alpha \right)\left(c_{a1}+c_{c1}\right)+2c_{b1}\alpha$$

The mean value of the fuzzy triangular number becomes:

$$E_{f}\left(C_{i}\right)=\frac{1}{2}\int_{0}^{1}\left(C_{1}\left(\alpha \right)+C_{1}\left(\alpha \right)\right)f\left(\alpha \right)d\alpha =\frac{1}{2}\int_{0}^{1}\left[\left(1-\alpha \right)\left(c_{a1}+c_{c1}\right)+2c_{b1}\alpha \right]2\alpha d\alpha \;E_{f}\left(C_{i}\right)=\left(c_{a1}+c_{c1}\right)\int_{0}^{1}\left(\alpha -\alpha^{2}\right)d\alpha +2c_{b1}\int_{0}^{1}\alpha^{2}d\alpha =\left(c_{a1}+c_{c1}\right)\frac{\alpha^{2}}{2}{│}_{0}^{1}-\left(c_{a1}+c_{c1}\right)\frac{\alpha^{3}}{3}{│}_{0}^{1}+2c_{b1}\frac{\alpha^{3}}{3}{│}_{0}^{1}\;E_{f}\left(C_{i}\right)=\frac{1}{6}\left(c_{a1}+c_{c1}\right)+\frac{2}{3}c_{b1}$$

## 4. Graphical Method for Solving Linear Programming Problem with Fuzzy Optimization: The Case of Two Tangible Assets

Two tangible assets are considered, ($A_{1},A_{2}$), to be purchased from a competitive market. Each of the two assets ($A_{1},A_{2}$) has a series of economic characteristics ($C_{ei}$), with $i=\bar{1,2,}$ as well as a series of technical characteristics ($C_{ti}$), with $i=\bar{1,2},$ which either are part of the mathematical model’s objective function, or are part of the company’s constraints, along with others resulting from the current activity, such as: budget for investments, budget for operating expenses of the tangible assets during their operation, etc.

The mathematical model of the linear programming problem with fuzzy variables becomes:

(23) $$\min z=\min f\left(x\right)=C_{a1}x_{1}+C_{a2}x_{2}$$

In this linear programming problem [52,53], $z=C_{a1}x_{1}+C_{a2}x_{2}$ represents the objective function that has to be minimized, the cost coefficients $C_{a1},\;C_{a2}$ represent the acquisition cost of the two assets ($A_{1},A_{2}$) modeled using triangular fuzzy numbers of the form $C_{a}=\left\{c_{a},\frac{\mu_{ca}}{c_{a}}\in C_{a}\right\}$ and $x_{1},$ $x_{2}$ represent the problem variables to be determined. The restrictions of the problem resulting from the constraints are of the form:

(24) $$\begin{array}{c} \{\begin{array}{c} C_{11}x_{1}+C_{12}x_{2}\leq B_{1}\\ C_{21}x_{1}+C_{22}x_{2}\leq B_{2}\\ \ldots \ldots \ldots \ldots \ldots \ldots \ldots \ldots \ldots \\ C_{n1}x_{1}+C_{n2}x_{2}\leq B_{n} \end{array}\\ x_{1},x_{2}\geq 0 \end{array}$$

The inequality $\sum_{j=1}^{n}C_{ij}x_{j}\leq B_{j}$ represents the restriction *i* of the company which may be, as mentioned before, the restriction of the assets acquisition criteria or the restriction of the activities carried out by the company [52,53]. The coefficients $C_{ij}\;$ with $i=\bar{1,n}\;\mathrm{and}\;j=\bar{1,m}$ are triangular fuzzy numbers of the form: $C=\left\{c,\frac{\mu_{c}}{c}\in C\right\},$ where $\mu_{c}:C\to \left[0,1\right]$ represented by intervals of variation of company constraints or criteria for the acquisition of tangible assets.

The vector $B=\left(\begin{array}{c} B_{1}\\ B_{2}\\ \ldots \\ B_{n} \end{array}\right)$ is also composed of triangular fuzzy numbers of the form $B=\left\{b,\frac{\mu_{b}}{b}\in B\right\},$ where $\mu_{b}:B\to \left[0,1\right]$ representing the maximum limits admissible for the linear programming problem restrictions (for further explanations related to vector B see [52,53]). The restrictions for variables $x_{1},x_{2}\geq 0$ are non-negative restrictions. The set $=\left\{x_{1},x_{2}\right\}$ that checks all the constraints of the linear programming problem is called the admissibility domain and the point ($x_{1},x_{2}$) is called the admissible point. Since the set *X* is formed by hyperplanes’ intersection (equality constraints) and closed half-space, it turns out that *X* is a polyhedron.

Solving the linear programming problem using the graphical method [53] thus implies determining the admissibility domain (of the problem’s solutions) by graphically representing the constraints of the problem, respectively determining the coordinate point ($x_{1},x_{2}$) that satisfies all the constraints and the objective function is of minimum/maximum after case. Each constraint of the linear programming problem of the form $C_{i1}x_{1}+C_{i2}x_{2}\leq B_{i}$ is thus represented by a half-space $C_{i1}x_{1}+C_{i2}x_{2}<B_{i}$ and a fuzzy equation of the form: $C_{i1}x_{1}+C_{i2}x_{2}=B_{i}$. Thus, each constraint involves solving a fuzzy equation and determining the corresponding half-space in which the solution of the constraint is found.

The next step is to solve the fuzzy equation of the form: $C_{i1}x_{1}+C_{i2}x_{2}=B_{i}.$ The solution of the fuzzy equation is obtained by giving values of $x_{1}=0$ and $x_{2}=0$ and by successively solving the fuzzy equations that are formed in variables $x_{1}$ and $x_{2}$.

If $x_{1}=0,$ then $C_{i2}x_{2}=B_{i},$ respectively the level sets for the triangular fuzzy number $C_{i2}=\left\{c_{i2},\frac{\mu_{c_{i2}}}{c_{i2}}\in C_{i2}\right\}$ for any $i=\bar{1,n}$ are of the form: ${\left[C_{i2}\right]}^{\alpha }=\left[C_{12}\left(\alpha \right);C_{22}\left(\alpha \right)\right]$ for any *α* ∈ [0,1] where:

(25) $$C_{12}\left(\alpha \right)=\left(c_{b2}-c_{a2}\right)\alpha +c_{a2}$$

(26) $$C_{22}\left(\alpha \right)=c_{c2}-\left(c_{c2}-c_{b2}\right)\alpha$$

For the triangular fuzzy number $B_{i}=\left\{b_{i},\frac{\mu_{b_{i}}}{b_{i}}\in B_{i}\right\}$ for any $i=\bar{1,n}\;,$ is of the form: ${\left[B_{i}\right]}^{\alpha }=\left[B_{1}\left(\alpha \right);B_{2}\left(\alpha \right)\right]$ for any *α* ∈ [0,1] where:

(27) $$B_{1}\left(\alpha \right)=\left(b_{b}-b_{a}\right)\alpha +b_{a}$$

(28) $$B_{2}\left(\alpha \right)=b_{c}-\left(b_{c}-b_{b}\right)\alpha$$

The solution of the fuzzy equation is obtained from the equals:

(29) $$\left(\left(c_{b2}-c_{a2}\right)\alpha +c_{a2}\right)x_{s}^{\alpha }=\left(b_{b}-b_{a}\right)\alpha +b_{a}$$

and respectively,

(30) $$\left(c_{c2}-\left(c_{c2}-c_{b2}\right)\alpha \right)x_{d}^{\alpha }=b_{c}-\left(b_{c}-b_{b}\right)\alpha$$

The solutions of the above fuzzy equation, namely the determination of the left part of the fuzzy number ($x_{s}^{\alpha }$) and the right part of the fuzzy number ($x_{d}^{\alpha }$), are obtained by applying the level set method and is of the form:

(31) $$x_{s}^{\alpha }=\frac{\left(b_{b}-b_{a}\right)\alpha +b_{a}}{\left(c_{b2}-c_{a2}\right)\alpha +c_{a2}}$$

and respectively,

(32) $$x_{d}^{\alpha }=\frac{b_{c}-\left(b_{c}-b_{b}\right)\alpha }{c_{c2}-\left(c_{c2}-c_{b2}\right)\alpha }$$

The solutions above verify the condition $x_{s}^{\alpha }<x_{d}^{\alpha }$ and for any $\alpha ,\alpha^{\prime }\in \left[0,1\right]$ with $\alpha <\alpha^{\prime }$, it turns out that $x_{s}^{\alpha }\leq x_{s}^{\alpha^{\prime }}$ and $x_{d}^{\alpha }\leq x_{d}^{\alpha^{\prime }}$. For *α* = 0 the crisp solution of the fuzzy equation is obtained as follows:

(33) $$x_{s}^{\alpha }=\frac{b_{a}}{c_{a2}}$$

and respectively,

(34) $$x_{d}^{\alpha }=\frac{b_{c}}{c_{c2}}$$

The fuzzy number resulting from solving the fuzzy equation has the coordinates $X=\left(x_{s}^{\alpha }=\frac{\left(b_{b}-b_{a}\right)\alpha +b_{a}}{\left(c_{b2}-c_{a2}\right)\alpha +c_{a2}};x_{d}^{\alpha }=\frac{b_{c}-\left(b_{c}-b_{b}\right)\alpha }{c_{c2}-\left(c_{c2}-c_{b2}\right)\alpha }\right)$ for any *α* ∈ [0,1] of the form $X=\left\{x,\frac{\mu_{x}}{x}\in X\right\},\;$ where $\mu_{x}:X\to \left[0,1\right].$ If $x_{2}=0$ results in the fuzzy $C_{i1}x_{1}=B_{i}$, respectively for the triangular fuzzy numbers $C_{i1}$ and respectively $B_{i}$ the following levels sets are obtained: $C_{i1}=\left\{c_{i1},\frac{\mu_{c_{i1}}}{c_{i1}}\in C_{i1}\right\}$ for any $i=\bar{1,n}$ are of the form: ${\left[C_{i1}\right]}^{\alpha }=\left[C_{11}\left(\alpha \right);C_{21}\left(\alpha \right)\right]$ for any *α* ∈ [0,1] where:

(35) $$C_{11}\left(\alpha \right)=\left(c_{b1}-c_{a1}\right)\alpha +c_{a1}$$

(36) $$C_{21}\left(\alpha \right)=c_{c1}-\left(c_{c1}-c_{b1}\right)\alpha$$

For the fuzzy triangular number $B_{i}=\left\{b_{i},\frac{\mu_{b_{i}}}{b_{i}}\in B_{i}\right\}$ for any $i=\bar{1,n}$ the level sets are of the form shown above, respectively: ${\left[B_{i}\right]}^{\alpha }=\left[B_{1}\left(\alpha \right);B_{2}\left(\alpha \right)\right]$ for any *α* ∈ [0,1] where [B_i] ^ *α* = [B_1 (*α*); B_2 (*α*)] for any *α* ∈ [0,1] where:

(37) $$B_{1}\left(\alpha \right)=\left(b_{b}-b_{a}\right)\alpha +b_{a}$$

(38) $$B_{2}\left(\alpha \right)=b_{c}-\left(b_{c}-b_{b}\right)\alpha$$

The solution of the fuzzy equation, as in the situation presented above, is obtained from the equals: $\left(\left(c_{b1}-c_{a1}\right)\alpha +c_{a1}\right)y_{s}^{\alpha }=\left(b_{b}-b_{a}\right)\alpha +b_{a}$ and respectively $\left(c_{c1}-\left(c_{c1}-c_{b1}\right)\alpha \right)y_{d}^{\alpha }=b_{c}-\left(b_{c}-b_{b}\right)\alpha .$ The fuzzy solutions of the above equation are of the form:

(39) $$y_{s}^{\alpha }=\frac{\left(b_{b}-b_{a}\right)\alpha +b_{a}}{\left(c_{b1}-c_{a1}\right)\alpha +c_{a1}}$$

(40) $$y_{d}^{\alpha }=\frac{b_{c}-\left(b_{c}-b_{b}\right)\alpha }{c_{c1}-\left(c_{c1}-c_{b1}\right)\alpha }$$

For α=0, the crisp solution of the fuzzy equation is:

(41) $$y_{s}^{\alpha }=\frac{b_{a}}{c_{a1}}$$

(42) $$y_{d}^{\alpha }=\frac{b_{c}}{c_{c1}}$$

**Figure 2:**
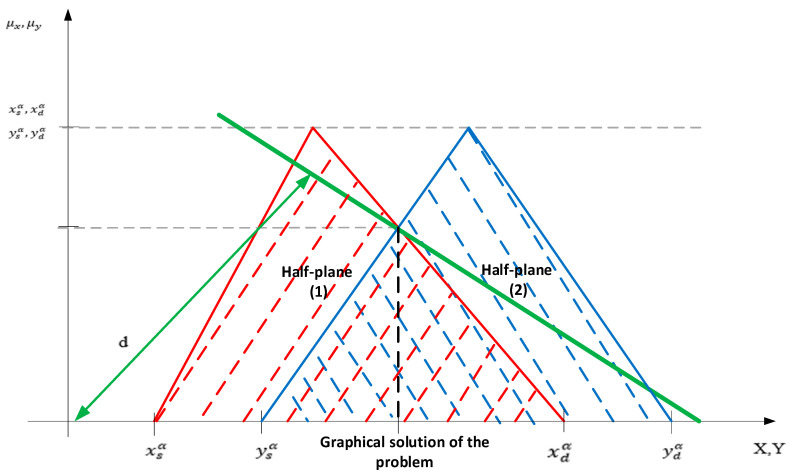
The graphical solution of the linear programming method.

Solving the above fuzzy equation results in the triangular fuzzy number $Y=\left(y_{s}^{\alpha }=\frac{\left(b_{b}-b_{a}\right)\alpha +b_{a}}{\left(c_{b1}-c_{a1}\right)\alpha +c_{a1}};y_{d}^{\alpha }=\frac{b_{c}-\left(b_{c}-b_{b}\right)\alpha }{c_{c1}-\left(c_{c1}-c_{b1}\right)\alpha }\right)$ with $\mu_{x}:X\to \left[0,1\right]$ for any *α* ∈ [0,1] of the form $Y=\left\{y,\frac{\mu_{y}}{y}\in Y\right\}$ where $\mu_{y}:Y\to \left[0,1\right]$. The half-space that is formed to identify the admissible solution of the linear programming problem is presented in Figure 2.

In order to check the half-space corresponding to the restriction of the linear programming problem, we verified if the solution of the linear programming problem is found in one of the lower or upper half-spaces. For this we substitute the coordinates of the origin O:(0,0) in the constraint of the problem of the form: $C_{i1}x_{1}+C_{i2}x_{2}\leq B_{i},$ from which will result: $C_{i1}0+C_{i2}0\leq B_{i}$, respectively $0\leq B_{i}$. The solution of the linear programming problem for which the objective function must be minimized, is identified in the lower half-space [53].

From the intersection of the half-spaces generated by the triangular fuzzy numbers, as a solution of the fuzzy equations (Figure 2), a fuzzy region is formed where the optimal solution of the linear programming problem is found. Its optimum is to determine the minimum or maximum distance as the objective function is the minimum or maximum between the line of the form Δ: $C_{a1}x_{1}+C_{a2}x_{2}=d,$ passing through origin *O:* (0,0) to the coordinate point $\left(x_{0},y_{0}\right)$.

(43) $$d\left(\left(x_{0},y_{0}\right),\Delta \right)=\frac{\left|C_{a1}x_{0}+C_{a2}x_{0}-d\right|}{\sqrt{C_{a1}^{2}+C_{a2}^{2}}}$$

By substituting the coordinates of the origin O: (0,0) in the above equation, we obtain: the coordinates of the origin O: (0,0) is obtained:

(44) $$d\left(\left(x_{0},y_{0}\right),\Delta \right)=\frac{\left|d\right|}{\sqrt{C_{a1}^{2}+C_{a2}^{2}}}$$

In conclusion, the solution of the linear programming problem depends on the value of *d,* which represents precisely the optimal function of the objective solution that respects all the restrictions resulting from the current activity of the company.

The coordinates of the fuzzy number result from the intersection of the lower half-space denoted by $Z=\left\{z,\frac{\mu_{z}}{z}\in Z\right\}$, where $\mu_{z}:Z\to \left[0,\alpha \right]$ is of the form: $Z=\left(z_{s}^{\alpha }=\frac{b_{a}}{c_{a1}};z_{d}^{\alpha }=\frac{b_{c}}{c_{c2}}\right)$ for values of *α* given by the equality:

(45) $$\frac{\left(b_{b}-b_{a}\right)\alpha +b_{a}}{\left(c_{b1}-c_{a1}\right)\alpha +c_{a1}}=\frac{b_{c}-\left(b_{c}-b_{b}\right)\alpha }{c_{c2}-\left(c_{c2}-c_{b2}\right)\alpha }$$

The above equality leads to the solution of the equation in *α* of the form:

(46) $$\left[\left(b_{b}-b_{a}\right)\alpha +b_{a}\right]\left[c_{c2}-\left(c_{c2}-c_{b2}\right)\alpha \right]=\left[b_{c}-\left(b_{c}-b_{b}\right)\alpha \right]\left[\left(c_{b1}-c_{a1}\right)\alpha +c_{a1}\right]$$

(47) $$\begin{array}{l} c_{c2}\left(b_{b}-b_{a}\right)\alpha -\left(b_{b}-b_{a}\right)\left(c_{c2}-c_{b2}\right)\alpha^{2}+b_{a}c_{c2}-b_{a}\left(c_{c2}-c_{b2}\right)\alpha \\ \;=b_{c}\left(c_{b1}-c_{a1}\right)\alpha +b_{c}c_{a1}-\left(b_{c}-b_{b}\right)\left(c_{b1}-c_{a1}\right)\alpha^{2}-c_{a1}\left(b_{c}-b_{b}\right)\alpha \end{array}$$

(48) $$\begin{array}{l} \alpha^{2}\left[\left(b_{b}-b_{a}\right)\left(b_{c}-b_{b}\right)\left(c_{c2}-c_{b2}\right)\left(c_{b1}-c_{a1}\right)\right]\\ \;+\alpha \left[c_{c2}\left(b_{b}-b_{a}\right)-b_{a}\left(c_{c2}-c_{b2}\right)-b_{c}\left(c_{b1}-c_{a1}\right)+c_{a1}\left(b_{c}-b_{b}\right)\right]\\ \;+b_{a}c_{c2}-b_{c}c_{a1}=0 \end{array}$$

We note that:

(49) $$\beta =\left(b_{b}-b_{a}\right)\left(b_{c}-b_{b}\right)\left(c_{c2}-c_{b2}\right)\left(c_{b1}-c_{a1}\right)$$

(50) $$\gamma =c_{c2}\left(b_{b}-b_{a}\right)-b_{a}\left(c_{c2}-c_{b2}\right)-b_{c}\left(c_{b1}-c_{a1}\right)+c_{a1}\left(b_{c}-b_{b}\right)$$

(51) $$\delta =b_{a}c_{c2}-b_{c}c_{a1}$$

With these notations the above equation becomes:

(52) $$\beta \alpha^{2}+\gamma \alpha +\delta =0$$

The solution of the above equation becomes:

(53) $$\alpha =\frac{-\gamma \pm \sqrt{\gamma^{2}-4\beta \delta }}{2\beta }$$

Under these conditions, the membership degree of fuzzy number $\mu_{z}:Z\to \left[0,\alpha \right]$, becomes $\mu_{z}:Z\to \left[0,\frac{-\gamma \pm \sqrt{\gamma^{2}-4\beta \delta }}{2\beta }\right]$. The fuzzy number Z is the solution of the linear programming problem for both maximum and minimum problems.

The graphical solution of the linear programming problem must be analyzed from the perspective of the intersection of the semi-spaces that determine the admissible solution of the problem. For constraints that determine the intersection of the upper semi-space, the solution of the linear programming problem is an intuitive fuzzy number, while the intersection of the upper semi-space with the lower semi-space generates a polyhedron in which the admissible solution is at one of the polyhedron peaks.

## 5. The Primal Simplex Algorithm with Fuzzy Variables for Minimization Problems: The Case with N-Tangible Assets

As it is known, a linear programming problem consists of minimizing or maximizing a linear objective function in relation to observing certain restrictions determined by the operating activity of the company, or as determined by the assets’ acquisition criteria. To base the primal simplex algorithm, the objective function is minimized. The linear programming problem has the canonical form [54]:

(54) $$\{\begin{array}{c} \min f\;\left(x\right)=\sum_{j=1}^{n}C_{aj}x_{j}\\ \sum_{j=1}^{n}C_{aj}x_{j}\;\leq B_{i}\\ j=\bar{1,n}\;and\;i=\bar{1,m}\\ x_{j}\geq 0\;with\;j=\bar{1,n} \end{array}$$

In the linear programming problem, $f\left(x\right)=\sum_{j=1}^{n}C_{aj}x_{j}$ represents the objective function minimized, the cost coefficients $C_{aj}$ represent the asset acquisition cost $A_{j},$ modeled using the triangular fuzzy numbers of the form $C_{a}=\left\{c_{a},\mu_{ca}/c_{a}\in C_{a}\right\}$ and $x_{j}$ represents the variables of the problem to be determined [52,54]. The problem variables are also fuzzy variables of the form: $X=\left\{x,\mu_{x}/x\in X\right\};X=\left(x_{1},x_{2}\right)$.

The inequality $\sum_{j=1}^{n}C_{ij}x_{j}\leq B_{j}$ represents the restriction *i* of the company which may be, as mentioned before, the restriction of the assets acquisition criteria, or the restriction of the activities carried out by the company. The coefficients $C_{ij}$ with $i=\bar{1,n}\;\mathrm{and}\;j=\bar{1,m\;}$ are triangular fuzzy numbers of the form: $C=\left\{c,\mu_{c}/c\in C\right\},$ where $\mu_{c}:C\to \left[0,1\right]$ is represented by the value ranges of the assets acquisition criteria or the restrictions of the company’s activity, but also of their membership degrees ($\mu_{c}$). The variables ($x_{j}$) are also fuzzy variables of the form: $X=\left\{x,\mu_{x}/x\in X\right\};X=\left(x_{1},x_{2}\right)$ to be determined.

The vector $B=\left(\begin{array}{c} B_{1}\\ B_{2}\\ \ldots \\ B_{n} \end{array}\right)$ is also composed of triangular fuzzy numbers of the form $B=\left\{b,\mu_{b}/b\in B\right\},$ where $\mu_{b}:B\to \left[0,1\right]$, represented by the maximum admissible limits for the restrictions of the linear programming problem. Restrictions for variables $x_{1},x_{2}\geq 0$ are non-negative restrictions. The standard form [52,54] of the linear programming problem is:

(55) $$\{\begin{array}{c} \min f\;\left(x\right)=\;\sum_{j=1}^{n}C_{aj}x_{j}\\ \sum_{j=1}^{n}C_{aj}x_{j}=B_{i}\\ j=\bar{1,n}\;and\;i=\bar{1,m}\\ x_{j}\geq 0\;with\;j=\bar{1,n} \end{array}$$

A linear programming problem is brought to the standard form by the following successive mathematical transformations known in the specialized literature [17,52,53,54]:

- A minimization problem is transformed into a maximization problem by changing the signs of the fuzzy coefficients from the respective objective function;
  (56) $$maxC_{a}^{T}x=-min\left[-C_{a}^{T}\right]x$$
- The sign of an inequality changes by multiplying it by (−1), respectively by multiplying the constraint with fuzzy variables and fuzzy coefficients by (−1).
- An inequality of the form $C_{i}^{T}x\leq B_{i}$ with $\left\{C_{i},B_{i}\right\}$} specific fuzzy sets, is written as an equality of the form $C_{i}^{T}x+Y=B_{i}$, with Y≥0, by adding the offset fuzzy variable $Y=\left\{y,\mu_{y}/y\in Y\right\}$, where $\mu_{y}:Y\to \left[0,1\right]$. An inequality of the form $C_{i}^{T}x\geq B_{i},\;$ with $\left\{C_{i},B_{i}\right\}$ specific fuzzy sets is written as an equality of the form $C_{i}^{T}x+Y=B_{i}$, with Y≥0, by subtracting the offset fuzzy $Y=\left\{y,\mu_{y}/y\in Y\right\}$, where $\mu_{y}:Y\to \left[0,1\right]$.
- An equality of the form $C_{i}^{T}x=B_{i}$ is transformed into two fuzzy inequalities of the form $C_{i}^{T}x\leq B_{i}$ and respectively $C_{i}^{T}x\geq B_{i}$.
  A negative fuzzy variable $x_{j}\leq 0$ is transformed into a positive fuzzy variable $x_{j}\geq 0$ by replacing with $-x_{j}.$ An unsigned fuzzy variable is replaced by the difference of two fuzzy variables $x_{j}=x_{j}^{\prime }-x_{j}^{\prime \prime },$ where $x_{j}^{\prime }\geq 0$ și $x_{j}^{\prime \prime }\geq 0$.

The above transformations lead to the standard mathematical model, with fuzzy coefficients $C_{ij}$ with $j=\bar{1,n}$ and $i=\bar{1,m}$ and also fuzzy variables to be determined, $x_{j}$ with $j=\bar{1,n.}$ The linear programming problem is thus summarized in determining the fuzzy vector $\in R^{n},$ which satisfies the condition $\min z=\sum_{j=1}^{n}C_{aj}x_{j}$ regarding the restrictions $\sum_{j=1}^{n}C_{ij}x_{j}\leq B_{i}$ and this is possible only if the mathematical model is brought to the standard fuzzy form. To solve the linear programming problem the following definitions are valid [47,48,52,54]:

- Definition 1: A fuzzy vector $X={\left(x_{1}\;x_{2}\ldots x_{n}\right)}^{T}\in R^{n}$ whose components satisfy all the constraints of the linear programming problem is called an admissible program or admissible fuzzy solution or possible fuzzy solution.
- Definition 2: An admissible solution of the form $X={\left(x_{1}\;x_{2}\ldots x_{n}\right)}^{T}\in R^{n}$ whose components minimize the objective function or, as the case may be, satisfy the condition imposed for the objective function, is called an optimal fuzzy program or optimal fuzzy solution.
- Definition 3: An admissible solution of the form $X={\left(x_{1}\;x_{2}\ldots x_{n}\right)}^{T}\in R^{n},\;$ whose column vectors $C^{j}$ corresponding to the nonzero components $x_{j}$ are linearly independent, is called te fuzzy basis program or fuzzy base solution.
- Definition 4: If a base program has nonzero fuzzy m-components (rank *C* = *m*), then the base program is called undesirable fuzzy. If a base program does not have null *m*-components (rank *C* = *m*) then the base program is called a fuzzy degenerate.
- Definition 5: The matrix B formed by *m* × *m* columns corresponding to the nonzero components of the fuzzy matrix *C*, of a non-degenerate base program *X* is called the fuzzy base of the program *X*.

Let be the linear programming problem mentioned above with fuzzy coefficients, with a non-degenerate basic program of the form $X^{*}={\left(x_{1}^{*},x_{2}^{*},x_{3}^{*},\ldots ,x_{m}^{*},0,0,0,\ldots 0\right)}^{T},$ where the main fuzzy variables are $\left(x_{1},x_{2},x_{3},\ldots ,x_{m}\right)$, while $\left(x_{m+1},x_{m+2},\ldots ,x_{n}\right)$ are the secondary fuzzy variables and the column vectors are the fuzzy numbers $\left(C_{1},C_{2},C_{3},\ldots ,C_{m}\right)$ that form the base *B* of the non-degenerate basic program $X^{*}$. Let $S=\left\{C^{m+1},C^{m+2},\ldots ,C^{n}\right\}$ represent the column vector of the fuzzy numbers that are not part of the base *B*. *S* is formed of non-basic fuzzy variables. It is noted with:

(57) $$X_{B}=\left(\begin{array}{c} x_{1}\\ x_{2}\\ \ldots \\ x_{m} \end{array}\right)\;and\;X_{S}=\left(\begin{array}{c} x_{m+1}\\ x_{m+2}\\ \ldots \\ x_{n} \end{array}\right)$$

(58) $$C_{aB}=\left(\begin{array}{c} C_{a1}\\ C_{a2}\\ \ldots \\ C_{am} \end{array}\right)\;\mathrm{and}\;C_{aS}=\left(\begin{array}{c} C_{am+1}\\ C_{am+2}\\ \ldots \\ C_{an} \end{array}\right)$$

(59) $$X^{T}=\left(x_{1}\;x_{2}\ldots \;x_{m}\;x_{m+1}\;x_{m+2}\ldots x_{n}\right)$$

We also note the following matrices as follows:

(60) $$C=\left(\begin{array}{cccc} C_{11} & C_{12} & \ldots & C_{1m}\\ C_{21} & C_{22} & \ldots & C_{2m}\\ \ldots & \ldots & \ldots & \ldots \\ C_{n1} & C_{n2} & \ldots & C_{nm} \end{array}\begin{array}{cccc} C_{1m+1} & C_{1m+2} & \ldots & C_{1n}\\ C_{2m+1} & C_{2m+2} & \ldots & C_{2n}\\ \ldots & \ldots & \ldots & \ldots \\ C_{nm+1} & C_{nm+2} & \ldots & C_{nn} \end{array}\right)$$

(61) $$B=\left(\begin{array}{cccc} C_{11} & C_{12} & \ldots & C_{1m}\\ C_{21} & C_{22} & \ldots & C_{2m}\\ \ldots & \ldots & \ldots & \ldots \\ C_{n1} & C_{n2} & \ldots & C_{nm} \end{array}\right);\;S=\left(\begin{array}{cccc} C_{1m+1} & C_{1m+2} & \ldots & C_{1n}\\ C_{2m+1} & C_{2m+2} & \ldots & C_{2n}\\ \ldots & \ldots & \ldots & \ldots \\ C_{nm+1} & C_{nm+2} & \ldots & C_{nn} \end{array}\right);\;B_{F}=\left(\begin{array}{c} B_{1}\\ B_{2}\\ \ldots \\ B_{n} \end{array}\right)$$

The matrices with the notations above have the coefficients $C_{ij}\;$ with $j=\bar{1,n}$ and $i=\bar{1,m},$ as well as the fuzzy coefficients $B_{i}$ with $i=\bar{1,n}$. The linear programming problem is of the form:

(62) $$\{\begin{array}{c} \min f\;\left(x\right)=\sum_{j=1}^{n}C_{aj}x_{j}\\ \sum_{j=1}^{n}C_{ij}x_{j}\;\leq B_{i}\\ j=\bar{1,n}\;and\;i=\bar{1,m}\\ x_{j}\geq 0\;with\;j=\bar{1,n} \end{array}$$

It is written in base *B* with fuzzy variables as follows:

(63) $$\{\begin{array}{c} min\;f\left(x\right)=C_{aB}X_{B}+C_{aS}X_{s}\\ BX_{B}+SX_{S}=B_{F}\\ X\geq 0 \end{array}$$

For the restriction part, we multiply the system of restrictions to the left and right with $B^{-1}$, thus obtaining:

(64) $$B^{-1}BX_{B}+SB^{-1}X_{S}=B^{-1}B_{F}$$

Since matrix *B* is an invertible matrix by multiplying *B* by $B^{-1}$ we obtain the unit matrix *I*, respectively $B^{-1}B=I$ so that the above relation is written as follows:

(65) $$X_{B}+SB^{-1}X_{S}=B^{-1}B_{F}\;\mathrm{or}\;X_{B}=B^{-1}B_{F}-SB^{-1}X_{S}$$

where $X_{B}$ are the fuzzy variables of base *B* also called the main fuzzy variables, while $X_{S}$ are the fuzzy variables of the matrix *S* also called the secondary fuzzy variables. The following notations are used:

(66) $$\bar{X_{B}}=B^{-1}B_{F}\;\mathrm{and}\;\bar{C_{ij}^{B}}=B^{-1}C_{j}$$

where $C_{j}$ represents the column *j* in the matrix *C* corresponding to the secondary variables $j\in J_{s}=\left\{j/C_{j}\in S\right\}$. In these conditions, the relationship $X_{B}=B^{-1}B_{F}-SB^{-1}X_{S}$ is rewritten as:

(67) $$X_{B}=\bar{X_{B}}-\overset{n}{\underset{j=1}{\sum }}\bar{C_{ij}^{B}}x_{j}$$

where $j\in J_{B}$; $J_{B}=\left\{i/C_{i}\in B\right\}$. The components above are written as follows:

(68) $$x_{i}=\bar{x_{j}^{B}}-\overset{n}{\underset{j=1}{\sum }}\bar{C_{ij}^{B}}x_{j}$$

Regarding the objective function, this is written using the main fuzzy variables $X_{B}\;$ and the secondary fuzzy variables $X_{S}$ as follows:

(69) $$f\left(x\right)=C_{a}^{T}X=\overset{n}{\underset{j=1}{\sum }}C_{j}x_{j}=\overset{m}{\underset{i\in J_{B}}{\sum }}C_{ai}x_{i}+\overset{n}{\underset{j\in J_{S}}{\sum }}C_{aj}x_{j}$$

(70) $$f\left(x\right)=\overset{m}{\underset{i\in J_{B}}{\sum }}C_{ai}\left(\bar{x_{j}^{B}}-\overset{n}{\underset{j=1}{\sum }}\bar{C_{ij}^{B}}x_{j}\right)+\overset{n}{\underset{j\in J_{S}}{\sum }}C_{aj}x_{j}$$

The above relation is written as:

(71) $$f\left(x\right)=\overset{m}{\underset{i\in J_{B}}{\sum }}C_{ai}\bar{x_{j}^{B}}-\overset{n}{\underset{j\in J_{S}}{\sum }}\left(\overset{m}{\underset{i\in J_{B}}{\sum }}C_{aj}\bar{C_{ij}^{B}}-C_{aj}\right)x_{j}$$

The following notations are used:

(72) $$\bar{Z^{B}}=\overset{m}{\underset{i\in J_{B}}{\sum }}C_{ai}\bar{x_{j}^{B}}=C_{aB}^{T}\bar{x_{B}}$$

(73) $$Z_{j}^{B}=\overset{m}{\underset{i\in J_{B}}{\sum }}C_{aj}\bar{C_{ij}^{B}}=C_{aB}^{T}C_{j}^{B}$$

In these conditions, the objective function is rewritten as:

(74) $$Z=\bar{Z^{B}}-\overset{n}{\underset{j\in J_{S}}{\sum }}\left(Z_{j}^{B}-C_{aj}\right)x_{j}$$

The linear programming problem with fuzzy variables and fuzzy coefficients, after these calculations and notations, is rewritten as follows:

(75) $$\{\begin{array}{c} Z=\bar{Z^{B}}-\sum_{j\in J_{S}}^{n}\left(Z_{j}^{B}-C_{aj}\right)x_{j}\\ X_{B}=\bar{X_{B}}-\sum_{j=1}^{n}\bar{C_{ij}^{B}}x_{j}\\ x_{i}=\bar{x_{j}^{B}}-\sum_{j=1}^{n}\bar{C_{ij}^{B}}x_{j}\\ x_{i}\geq 0;i\in \bar{1,n} \end{array}$$

For the linear programming problem with fuzzy variables and fuzzy coefficients, for the foundation of the primal simplex algorithm the following theorems are valid [38,39,48,50,51]:

Theorem 1: If the fuzzy difference of the objective function is negative, respectively $Z_{j}^{B}-C_{aj}\leq 0$ for any $j\in J_{S},$ then the program is optimal and the basic solution $\bar{X_{B}}=B^{-1}B_{F}$ and $\bar{X_{S}}=SB^{-1}X_{S}=0$ is the optimal solution to the linear programming problem.

Demonstration: For any program of the linear programming problem, the condition for the fuzzy variables $x_{j}\geq 0$ must be met as a condition of non-negativity which means that:

$$\overset{n}{\underset{j\in J_{S}}{\sum }}\left(Z_{j}^{B}-C_{aj}\right)x_{j}\leq 0$$

In conditions of $Z_{j}^{B}-C_{aj}\leq 0$ and $x_{j}\geq 0.$ From the objective function relation, we obtain that:

$$Z=Z^{B}-\overset{n}{\underset{j\in J_{S}}{\sum }}\left(Z_{j}^{B}-C_{aj}\right)x_{j}$$

It is known that the term: $\sum_{j\in J_{S}}^{n}\left(Z_{j}^{B}-C_{aj}\right)x_{j}\leq 0$, which determines the objective function value.

$$Z=\bar{Z^{B}}-\overset{n}{\underset{j\in J_{S}}{\sum }}\left(Z_{j}^{B}-C_{aj}\right)x_{j}\geq 0$$

The solution thus determined is optimal and causes an increase in the objective function *Z*.

Theorem 2: If there is a fuzzy variable $x_{k}\in J_{s}$ for which the difference $Z_{j}^{B}-C_{aj}>0$, then the solution generated by the basic program $\bar{X_{B}}=B^{-1}B_{F}$ and $\bar{X_{S}}=SB^{-1}X_{S}=0$ is not optimal and is improved if the fuzzy variable takes positive values $x_{k}>0$.

Demonstration: It is supposed that the value of the secondary fuzzy variable reaches the value $x_{k}^{*}$ for which we have $x_{k}^{*}>0$ and the fuzzy variable $x_{k}>x_{k}^{*}$. For the two fuzzy variables we determine the value of the objective function. The objective function value for $x_{k}>0$ is of the form:

$$Z=\bar{Z^{B}}-\overset{n}{\underset{j\in J_{S}}{\sum }}\left(Z_{j}^{B}-C_{aj}\right)x_{j}$$

For $x_{k}^{*}>0,$ the value of the objective function changes and becomes the form of:

$$Z^{*}=\bar{Z^{B}}-\overset{n}{\underset{j\in J_{S}}{\sum }}\left(Z_{j}^{B}-C_{aj}\right)x_{j}^{*}\to Z^{*}<Z$$

In conclusion, increasing $x_{k}^{*}>0$ results in an improvement in the value of the *Z* function since $Z^{*}<Z$. The program $Z^{*}$ is not a basic program because it has *m* + 1 strictly positive components, respectively $x_{i}$ with $i\in J_{B}$ and $x_{k}.$ When $x_{k}>0$ grows, the question is how much $x_{k}$ can grow so that it remains in the set of basic solutions.

Theorem 3: If there is a fuzzy variable $x_{k},k\in J_{S},$ such that the difference $Z_{j}^{B}-C_{aj}>0$ and all $C_{ij}\leq 0$ for any $j\in J_{B}$, then the linear programming problem has an infinite optimal.

Demonstration: We know that:

$$X_{B}=\bar{X_{B}}-\overset{n}{\underset{j=1}{\sum }}\bar{C_{ij}^{B}}x_{j}$$

The above relation written in components is of the form:

$$x_{i}=\bar{x_{i}}-\bar{C_{ik}}x_{k}\forall \;i\in J_{B}$$

With $x_{j}=0$ for any $j\in J_{S}$ and $x_{k}\geq 0$.

The value of the objective function under these conditions becomes:

$$Z=\bar{Z^{B}}-\left(Z_{k}^{B}-C_{k}\right)x_{k}^{*}$$

$$\underset{x_{k}^{*}\to \infty }{\lim}\bar{Z^{B}}-\left(Z_{k}^{B}-C_{k}\right)x_{k}^{*}=-\infty$$

So, the objective function is of the form:

$$Z=\bar{Z^{B}}-\overset{n}{\underset{j\in J_{S}}{\sum }}\left(Z_{j}^{B}-C_{aj}\right)x_{j}\to -\infty$$

In conclusion, it can be stated that for large values of fuzzy variables $x_{k}^{*}\to \infty$, the value of the objective function *Z* is very small.

Theorem 4: If the fuzzy difference in the objective function is positive $Z_{j}^{B}-C_{aj}>0$ and there is $C_{ik}\geq 0$, then the value of the fuzzy variable $x_{k}$ increases until it reaches the value:

(76) $$x_{k}=\underset{j\in J_{B}}{\min}\left\{\frac{x_{j}}{C_{ij}}\right\},\;C_{ik}\geq 0$$

Demonstration: We know that the main fuzzy variables are written as:

$$X_{B}=\bar{X_{B}}-\overset{n}{\underset{j=1}{\sum }}\bar{C_{ij}^{B}}x_{j}$$

Also, the main fuzzy variables are written as components:

$$x_{i}=\bar{x_{i}}-\bar{C_{ik}}x_{k}\forall \;i\in J_{B}$$

$$\bar{x_{i}}-\bar{C_{ik}}x_{k}=0$$

$$x_{k}=\frac{x_{i}}{C_{ik}}\;\forall \;i\in J_{B}$$

If $C_{ik}>0$, then we need $x_{k}=\underset{j\in J_{B}}{\min}\left\{\frac{x_{j}}{C_{ij}}\right\}$ to maintain the value of $x_{i}$ in the set of base programs.

In conclusion, increasing the value of the fuzzy variable $x_{k}$ can occur until $x_{k}\leq \frac{x_{j}}{C_{ij}}\;\left(\forall \right)\;i\in J_{B}$ and $C_{ik}>0$. For the obtained value of $x_{k}$, the value of $x_{i}$ is canceled and a new program is obtained in which the basic variables are $x_{k}$ and respectively $x_{i},$ with $i\in J_{B}-\left\{k\right\}.$ Corresponding to the increase of $x_{k}\leq \frac{x_{j}}{C_{ij}}$, a decrease in the value of the objective function is obtained by the value $\frac{x_{j}}{C_{ij}}\left(Z_{j}^{B}-C_{aj}\right)$.

Based on the above theorems, the primal simplex algorithm [7,52,53,54] with fuzzy coefficients and fuzzy variables, for minimum problems, as established by the mathematical model of the linear programming problem, involves the following steps:

- Step 1: Determine an admissible primal base B and determine its specific sizes, namely: $\bar{X_{B}}$; $\bar{Z_{B}}$; $\bar{C_{ij}^{B}}$; $Z_{j}^{B}-C_{aj}$. Determining these specific sizes is necessary to determine the value of the objective function Z and the solution of problem $X_{B},$ as:
  (77) $$Z=\bar{Z^{B}}-\sum_{j\in J_{S}}^{n}\left(Z_{j}^{B}-C_{aj}\right)x_{j}$$
  (78) $$X_{B}=\bar{X_{B}}-\sum_{j=1}^{n}\bar{C_{ij}^{B}}x_{j}$$
- Step 2: Analyze all the fuzzy differences resulting from the objective function following the rules of the fuzzy differences operator $Z_{j}^{B}-C_{aj}$ (the entry criterion). There are the following situations:
  ○ 2.1: If all fuzzy differences are negative $Z_{j}^{B}-C_{aj}\leq 0,\;$ then the program is optimal. Subtracting the fuzzy numbers $Z_{j}^{B}$ and $C_{aj}$ is done as follows:
    (79) $$Z_{j}^{B}-C_{aj}=\left[z_{ja1}^{B},z_{jb1}^{B}\right]-\left[c_{aj1},c_{aj2}\right]=\left[z_{ja1}^{B}-c_{aj1},z_{jb1}^{B}-c_{aj2}\right]$$
  ○ 2.2: If there is at least one index $j\in J_{S},$ for which the fuzzy difference is $Z_{j}^{B}-C_{aj}>0,$ then $k\in J_{S}$ is determined for which:
    (80) $$Z_{k}^{B}-C_{ak}=max\left(Z_{j}^{B}-C_{aj}\right)$$
- Step 3: Establish the vector for the base exit (the base exit criterion), according to the following algorithm:
  ○ 3.1: If all the fuzzy coefficients are negative $C_{ij}\leq 0$ and the fuzzy difference is positive $Z_{j}^{B}-C_{aj}>0$ for any $j\in J_{B}$, then the linear programming problem has an infinite optimal according to Theorem 3 presented above.
  ○ 3.2: If all the fuzzy coefficients are positive $C_{ij}>0$ and the fuzzy difference is positive $Z_{j}^{B}-C_{aj}>0$, for any $j\in J_{B}$, then the vector $x_{l}$ is chosen which replaces the vector $x_{k}$ and determines a new allowable basis $\tilde{B}$ with the value:
    (81) $$x_{l}=\underset{j\in J_{B}}{\min}\left\{\frac{x_{i}}{C_{ik}}\right\};\;C_{ik}>0$$
- Step 4: The vector $x_{l}$ is replaced by the vector $x_{k}$ in base *B* determined as the new allowable base $\tilde{B}$ for which $\bar{X_{\tilde{B}}}$; $\bar{Z_{\tilde{B}}}$; $\bar{C_{ij}^{\tilde{B}}}$; $Z_{j}^{\tilde{B}}-C_{aj}$. The determination of specific values $\bar{X_{\tilde{B}}}$; $\bar{Z_{\tilde{B}}}$; $\bar{C_{ij}^{\tilde{B}}}$; $Z_{j}^{\tilde{B}}-C_{aj}\;$ for the new allowable base $\tilde{B}$ is made according to the values of the allowable base *B* as follows. We know that the components of the main variable are written as follows:
  (82) $$x_{i}=\bar{x_{j}^{B}}-\overset{n}{\underset{j=1}{\sum }}\bar{C_{ij}^{B}}x_{j}$$

For *i* = *l* we obtain the expression by components of the main variable values as follows:

(83) $$x_{l}=\bar{x_{l}^{B}}-\overset{n}{\underset{j\in S-\left\{k\right\}}{\sum }}C_{lj}^{B}x_{j}-C_{ik}^{B}x_{k}$$

From the above relationship, because $C_{ik}^{B}\neq 0$ we get its value $\left(x_{k}\right)$ of the form:

(84) $$x_{k}=\frac{\bar{x_{l}^{B}}-\sum_{j\in S-\left\{k\right\}}^{n}C_{lj}^{B}x_{j}-x_{l}}{C_{ik}^{B}}$$

(85) $$x_{k}=\frac{\bar{x_{l}^{B}}}{C_{ik}^{B}}-\frac{\sum_{j\in S-\left\{k\right\}}^{n}C_{lj}^{B}x_{j}}{C_{ik}^{B}}-\frac{x_{l}}{C_{ik}^{B}}$$

Since the variable $x_{k}$ becomes a basic variable we also write that:

(86) $$x_{k}=\bar{x_{k}^{\tilde{B}}}-\overset{n}{\underset{j=1}{\sum }}\bar{C_{ik}^{\tilde{B}}}x_{j}$$

From the calculation relation for the fuzzy vector $x_{k}$, the specific calculation relations for $\bar{x_{k}^{\tilde{B}}}$ and $\bar{C_{ik}^{\tilde{B}}}$ are obtained as follows:

(87) $$\bar{x_{k}^{\tilde{B}}}=\frac{\bar{x_{l}^{B}}}{C_{ik}^{B}}și\;\bar{C_{ik}^{\tilde{B}}}=\frac{C_{lj}^{B}}{C_{ik}^{B}}$$

On the other hand, from: $x_{l}=\bar{x_{l}^{B}}-\overset{n}{\underset{j\in S-\left\{k\right\}}{\sum }}C_{lj}^{B}x_{j}-C_{ik}^{B}x_{k}$ and $x_{k}=\frac{\bar{x_{l}^{B}}-\sum_{j\in S-\left\{k\right\}}^{n}C_{lj}^{B}x_{j}-x_{l}}{C_{ik}^{B}}$ we obtain that:

(88) $$x_{l}=\bar{x_{l}^{B}}-\overset{n}{\underset{j\in S-\left\{k\right\}}{\sum }}C_{lj}^{B}x_{j}-C_{ik}^{B}\left(\frac{\bar{x_{l}^{B}}-\sum_{j\in S-\left\{k\right\}}^{n}C_{lj}^{B}x_{j}-x_{l}}{C_{ik}^{B}}\right)$$

Since the variable $x_{l}$ becomes a basic variable we also write that:

(89) $$x_{l}=\bar{x_{l}^{\tilde{B}}}-\overset{n}{\underset{j=1}{\sum }}\bar{C_{il}^{\tilde{B}}}x_{j}$$

Under these conditions, the computation relation for $\bar{x_{i}^{\tilde{B}}}$ and $\bar{C_{il}^{\tilde{B}}}$ are obtained, which is determined as follows:

(90) $$\bar{x_{i}^{\tilde{B}}}=\bar{x_{i}^{B}}-\frac{C_{ik}^{B}\bar{x_{l}^{B}}}{C_{ik}^{B}}\;și\;\bar{C_{il}^{\tilde{B}}}=C_{ij}^{B}-\frac{C_{ik}^{B}C_{lj}^{B}}{C_{ik}^{B}}$$

For the new admissible basis $\tilde{B}$ the calculation relation that is the basis for determining the value of the objective function is obtained as follows:

(91) $$Z^{\tilde{B}}=\bar{Z_{k}^{B}}-\left(Z_{k}^{B}-C_{k}\right)\frac{\bar{x_{l}^{B}}}{C_{ik}^{B}}$$

(92) $$Z_{j}^{\tilde{B}}-C_{j}=Z_{j}^{B}-C_{j}-\left(Z_{k}^{B}-C_{k}\right)\frac{C_{lj}^{B}}{C_{ik}^{B}}$$

In conclusion, it can be stated that the specific elements of the new admissible basis $\tilde{B}$ can be written according to the specific elements of the admissible basis *B*, according to the algorithm that was presented previously. If the solution is not optimal, the primal fuzzy algorithm from Step 2 is resumed. For the ordering of the manual calculations, simplex tables [7,52,53,54] are used in the practical application of the simplex algorithm, with *m* + 1 lines and *n* + 1 columns, for each admissible calculation base. The first *m*-lines corresponding to the allowable basis *B* contain the components of the column vectors $X^{B}$ and $C_{j}^{B}$ with 1≤j≤n and the last line contains the values $Z^{B}$ and $Z_{j}^{B}-C_{j}$ with 1≤j≤n.

The fuzzy simplex table is presented in Table 1.

The values for $Z^{B}$ are obtained according to the calculation formula $Z^{B}\left(B_{F}\right)=\sum_{i=1}^{n}C_{i}^{B}B_{i}$ and similarly the value of $Z^{B}$ is obtained for the fuzzy variables $x_{i}$ following the rule of fuzzy multiplication, according to the computation relation $Z^{B}\left(x_{i}\right)=\sum_{i=1}^{n}C_{i}^{B}C_{ij}$ with $j=\bar{1,n}$ and $i=\bar{1,m}$. The values for $Z_{j}^{B}-C_{j}$ are obtained according to the relation Z$Z_{j}^{B}-C_{j}=Z^{B}\left(x_{i}\right)-C_{ai}$ after deduction from the value of $Z^{B}\left(x_{i}\right)$ the value related to the fuzzy coefficient of the objective function $C_{ai}$.

**Figure 3:**
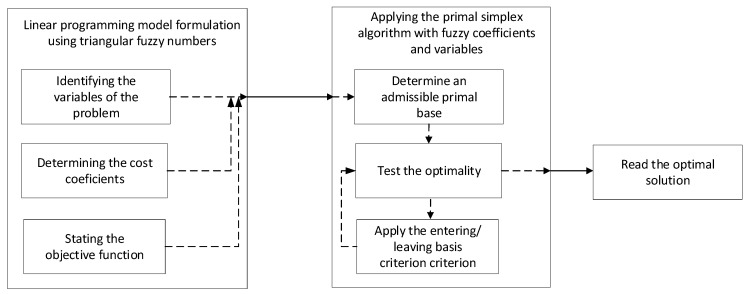
The flow chart of problem solving using simplex algorithms with fuzzy coefficients.

In order to perform an iteration, to move from one simplex table to another [7,52,53,54], we must take into account the vector $x^{k}$ entering the base, as well as the vector $x^{l}$ leaving the base, since at the intersection of the vector entering the base $x^{k}$ and the vector that comes out of the base $x^{l}$ is the element $C_{kl}$ called pivot. For the simplex table [7,52,53,54], in order to obtain the fuzzy components, the following rules are observed:

Rule 1: All the elements in the line with the pivot $C_{kl}$ are divided by the pivot;
Rule 2: All the elements in the pivot column become zero, except the pivot whose value becomes 1;
Rule 3: All the other elements of the table are obtained according to the rule of the rectangle;
  (93) $$\tilde{C_{ij}}=C_{ij}-\frac{C_{lj}C_{ik}}{C_{kl}}$$
  The simplex table is prepared for each iteration of the calculation until the differences $Z_{j}^{B}-C_{j}=Z^{B}\left(x_{i}\right)-C_{ai}\leq 0$ in which, according to Theorem 1, the program is optimal [7,52,53,54]. The entire process presented above is summarized in the flowchart in Figure 3.

## 6. Case Study on the Application of the Primal Simplex Algorithm with Fuzzy Coefficients

For the application of the primal simplex algorithm, three tangible assets ($A_{1},A_{2},A_{3}$) were taken into account, which specify that they are characterized by a series of acquisition criteria but also by constraints resulting from the current activity of the company. The acquisition criteria, consisting mainly of the purchase price or the operating expenses of the three tangible assets, are presented in the form of fuzzy triangular numbers with a single value range. The constraints of the company’s activity are represented by the financial constraints resulting from the budgets allocated by the company for different categories of activities or are represented by technical constraints. Regardless of their nature, the constraints of the company’s activity are also presented in the form of fuzzy triangular numbers. Table 2 presents the acquisition criteria, as well as the constraints resulting from their activity.

Based on the data presented in the previous table, it is required to establish, with the help of the primal simplex algorithm, the quantities of tangible assets to be purchased from the market so that the acquisition cost of the tangible assets is minimized and the constraints of the linear programming problem are met. The mathematical model for applying the primal simplex algorithm is established as follows:

- The objective function with fuzzy coefficients and variables is of the form:
  (94) $$f\left(x\right)=\left(1000,\;1500\right)x_{1}+\left(2000,\;3000\right)x_{2}+\left(5000,\;10,000\right)x_{3}\to min$$
- The constraints of the linear programming problem with fuzzy coefficients are:
  (95) $$\{\begin{array}{c} \left(1000,\;1500\right)x_{1}+\left(2000,\;3000\right)x_{2}+\left(5000,\;10,000\right)x_{3}\leq \left(100,000,\;200,000\right)\\ 10x_{1}+50x_{2}+100x_{3}\leq \left(5000,\;10,000\right)\\ \left(100,\;150\right)x_{1}+\left(200,\;300\right)x_{2}+\left(500,\;1000\right)x_{3}\leq \left(25,000,\;50,000\right) \end{array}$$
- The non-negativity conditions of the linear programming problem are of the form:
  $$x_{1}\geq 0,x_{2}\geq 0,\;x_{3}\geq 0;\;$$

In the linear programming problem the objective function formed by the acquisition costs of the three assets ($A_{1},A_{2},\;A_{3}$) must be minimized f (x) → min, while the fuzzy variables $x_{1},x_{2},x_{3}$ represent the quantities of tangible assets to be acquired from the market and must be determined. According to the mathematical model of the linear programming problem, the next step is to bring the problem to the standard form, that occurs by introducing the fuzzy offset variables $x_{4},x_{5},x_{6}$. Bringing the problem to the standard form has the following form:

- The objective function with fuzzy deviation variables is set as follows:
  (96) $$f\left(x\right)=\left(1000,\;1500\right)x_{1}+\left(2000,\;3000\right)x_{2}+\left(5000,\;10,000\right)x_{3}+0x_{4}+0x_{5}+0x_{6}\to min$$
- The constraints of the linear programming problem with the help of the fuzzy offset variables are established as follows:
  (97) $$\{\begin{array}{c} \left(1000,\;1500\right)x_{1}+\left(2000,\;3000\right)x_{2}+\left(5000,\;10,000\right)x_{3}+x_{4}=\left(100,000,\;200,000\right)\\ 10x_{1}+50x_{2}+100x_{3}+x_{5}=\left(5000,\;10,000\right)\\ \left(100,\;150\right)x_{1}+\left(200,\;300\right)x_{2}+\left(500,\;1000\right)x_{3}+x_{6}=\left(25,000,\;50,000\right) \end{array}$$
- The non-negativity conditions for the mathematical model, completed with the help of the offset variables are of the form: $x_{1}\geq 0,$ $x_{2}\geq 0,\;x_{3}\geq 0,x_{4}\geq 0,$ $x_{5}\geq 0,\;x_{6}\geq 0$.

The first simplex table corresponding to the admissible starting base *B* resulting from the first iteration of the primal simplex algorithm is presented in Table 3.

The other iterations of the primal simplex algorithm have the simplex table drawn up according to the rules presented in Section 5. The admissible starting base *B* of linear programming problem consists of the main fuzzy variables:

(98) $$\{\begin{array}{c} x_{4}=\left(100,000,\;200,000\right)\\ x_{5}=\left(5000,\;10,000\right)\\ x_{6}=\left(25,000,\;50,000\right) \end{array}$$

The secondary fuzzy variables of the initial admissible base are:

(99) $$\{\begin{array}{c} x_{1}=\left(0,\;0\right)\\ x_{2}=\left(0,\;0\right)\\ x_{3}=\left(0,\;0\right) \end{array}$$

The calculations corresponding to $Z^{B}$ are shown below as well as the calculations corresponding to $Z_{j}^{B}-C_{j}.$

(100) $$Z^{B}\left(B_{F}\right)=\left(0,\;0\right)\left(100,000,\;200,000\right)+\left(0,\;0\right)\left(5000,\;10,000\right)+\left(0\;,0\right)\left(25,000,\;50,000\right)$$

(101) $$Z^{B}\left(x_{1}\right)=\left(0,\;0\right)\left(1000,\;1500\right)+\left(0,\;0\right)\left(10\right)+\left(0,\;0\right)\left(100,\;150\right)=\left(0,\;0\right)$$

(102) $$Z^{B}\left(x_{2}\right)=\left(0,\;0\right)\left(2000,\;3000\right)+\left(0,\;0\right)\left(50\right)+\left(0,\;0\right)\left(200,\;300\right)=\left(0,\;0\right)$$

(103) $$Z^{B}\left(x_{3}\right)=\left(0,\;0\right)\left(5000,\;10,000\right)+\left(0,\;0\right)\left(100\right)+\left(0,\;0\right)\left(500,\;1000\right)=\left(0,\;0\right)$$

The values for $Z_{j}^{B}-C_{j}$ are obtained as:

(104) $$Z_{1}^{B}-C_{1}=\left(0,\;0\right)-\left(1000,\;1500\right)=\left(-1000,-1500\right)$$

(105) $$Z_{2}^{B}-C_{2}=\left(0,\;0\right)-\left(2000,\;3000\right)=\left(-2000,-3000\right)$$

(106) $$Z_{3}^{B}-C_{3}=\left(0,\;0\right)-\left(5000,\;10,000\right)=\left(-5000,-10,000\right)$$

After all the iterations, the fuzzy values for the quantities of tangible assets that should be purchased are obtained as follows:

(107) $$x_{1}=\left(2.73,\;3.33\right)$$

(108) $$x_{2}=\left(5.47,\;6.67\right)$$

(109) $$x_{3}=\left(16.67,\;17.91\right)$$

The value for the objective function is obtained as:

(110) $$\begin{array}{l} f\left(x\right)\\ =\left(1000,1500\right)\left(2.73,3.33\right)+\left(2000,3000\right)\left(5.47,6.67\right)+\left(5000,10,000\right)\left(16.67,17.91\right)\\ +0x_{5}+0x_{6}\\ =\left(min\left(2730,3330,4095,4995\right);max\left(2730,3330,4095,4995\right)\right)\\ +\left(min\left(10,940,13,340,16,410,20,010\right);max\left(10,940,13,340,16,410,20,010\right)\right)\\ +\left(min\left(83,350,89,550,166,700,170,910\right);max\left(83,350,89,550,166,700,170,910\right)\right) \end{array}$$

(111) f(x)=(2730, 4995)+(10,940, 20,010)+(83,350, 170,910)=(100,020, 195,515)

The economic interpretation of the obtained results indicates that the company is able to purchase within the limit of the budget allocated for the investment activity, respectively between ($100,000; $200,000) the quantities of approximately $x_{1}$ = (2.73, 3.33) of the first asset $A_{1}$, $x_{2}$ = (5.47, 6.67) from asset $A_{2}$ and $x_{3}$ = (16.67, 17.91) from asset $A_{3}$. The limits resulting from the linear programming calculations for assets quantities ($A_{1},A_{2},A_{3}$) assure the company that all the restrictions imposed by its current activity are respected and that the objective function is minimized.

## 7. Concluding Remarks

This paper studies the substantiation of the investment decision for the acquisition of tangible assets necessary for the company’s activity by using linear programming, respectively, by implementing two methods that underpin linear programming, namely: the graphical method and the primal simplex algorithm. Both the graphical method and the primal simplex algorithm are based on decision variables, coefficients from the objective function, but also from the matrix of constraints in the form of triangular fuzzy numbers. The primal simplex algorithm was presented theoretically and practically implemented by using fuzzy variables and fuzzy coefficients, which ultimately obtained the results of the linear programming problem in the form of fuzzy variables that underpin the investment decision-making process of the companies. The major advantage of these results is that they are presented as value ranges. From these value ranges, the company can select any of the results values as they satisfy two basic requirements, namely: minimize/maximize the objective function and satisfy the basic requirements regarding the constraints resulting from the activity of the company.

The management tool created with the help of linear programming and with the help of the fuzzy variables and fuzzy coefficients solves complex decision problems for companies, by ensuring the combination of three fundamental elements underlying the acquisition of assets, namely: the available financial resources of the company, which are limited in character; the constraints of the current activity determined in particular by the technological flow, but also by the budgets allocated on activities; and the economic performance of the assets acquired.

The fuzzy optimization in linear programming problems for substantiating the investment decisions of the company is both useful and necessary as it allows the decisions selection that respond to the fundamental requirements mentioned in this paper, namely the assets economic performance, the economic benefits generated during their operation period, and the company’s constraints. The algorithm can be extended to other specific situations resulting from the substantiation of the investment decisions, according to the specific needs of the company.

Future research directions will include the development of a new financial risk mitigation model, using the primal simplex algorithm and neutrosophic fuzzy numbers with applications on financial markets.

## Figures and Tables

**Table 1:** The simplex table corresponding to base *B*.

The Start Admissible Base (*B*)	The Fuzzy Coefficients from Base *B*	The Objective Function Coefficients	$C_{a1}$	$C_{a2}$		$C_{an}$
		Variable Values $B_{F}$	$x_{1}$	$x_{2}$	…	$x_{n}$
$x_{1}^{B}$	$C_{1}^{B}$	$B_{1}$	$C_{11}$	$C_{21}$	…	$C_{n1}$
$x_{2}^{B}$	$C_{2}^{B}$	$B_{2}$	$C_{21}$	$C_{22}$	…	$C_{2n}$
…	…	…	…	…	…	…
$x_{n}^{B}$	$C_{n}^{B}$	$B_{n}$	$C_{n1}$	$C_{n2}$	…	$C_{nn}$
$Z^{B}$	***	$Z^{B}\left(B_{F}\right)$	$Z^{B}\left(x_{1}\right)$	$Z^{B}\left(x_{2}\right)$	…	$Z^{B}\left(x_{n}\right)$
$Z_{j}^{B}-C_{j}$	***	***	$Z^{B}\left(x_{1}\right)-C_{a1}$	$Z^{B}\left(x_{2}\right)-C_{a2}$	…	$Z^{B}\left(x_{n}\right)-C_{an}$

**Table 2:** Fuzzy numbers values for acquisition criteria and constraints.

Criterion	Criterion Type: Acquisition/Constraint Resulting from Company’s Activity	Notation	The Value for Asset $\left(A_{1}\right)$	The Value for Asset $\left(A_{2}\right)$	The Value for Asset $\left(A_{3}\right)$
Acquisition cost	Acquisition criteria	$C_{a}\left(A_{i}\right)$	$\left(\begin{array}{c} \$1000,\\ \$1500 \end{array}\right)$	$\left(\begin{array}{c} \$2000,\\ \$3000 \end{array}\right)$	$\left(\begin{array}{c} \$5000\\ \$10,000 \end{array}\right)$
The allocated budget for the tangible assets acquisition	Activity constraint	$B_{inv}$	($100,000, $200,000)		
The mounting surface/asset	Activity constraint	$S_{m}\left(A_{i}\right)$	10 m^2^	50 m^2^	100 m^2^
The total surface for mounting	Activity constraint	$St_{m}$	$\left(5,000\;m^{2},\;10,000\;m^{2}\right)$		
The operating expenses	Acquisition criteria	$Ch_{f}\left(A_{i}\right)$	$\left(\begin{array}{c} \$100,\;\\ \$150 \end{array}\right)$	$\left(\begin{array}{c} \$200,\;\\ \$300 \end{array}\right)$	$\left(\begin{array}{c} \$500,\;\\ \$1000 \end{array}\right)$
The allocated budget for the operating expenses	Activity constraint	$BCh_{f}$	($25,000, $50,000)		

**Table 3:** Simplex table corresponding to the admissible starting base.

The Admissible Starting Base (*B*)	The Fuzzy Coefficients from Base *B*	The Coefficients of the Objective Function	$\left(\begin{array}{c} 1000,\\ 1500 \end{array}\right)$	$\left(\begin{array}{c} 2000,\\ 3000 \end{array}\right)$	$\left(\begin{array}{c} 5000,\\ 10,000 \end{array}\right)$	(0,0)	(0,0)	(0,0)
		$B_{F}$	$x_{1}$	$x_{2}$	$x_{3}$	$x_{4}$	$x_{5}$	$x_{6}$
$x_{4}^{B}$	(0,0)	$\left(\begin{array}{c} 100,000,\\ 200,000 \end{array}\right)$	$\left(\begin{array}{c} 1000,\\ 1500 \end{array}\right)$	$\left(\begin{array}{c} 2000,\\ 3000 \end{array}\right)$	$\left(\begin{array}{c} 5000,\\ 10,000 \end{array}\right)$	(1,1)	(0,0)	(0,0)
$x_{5}^{B}$	(0,0)	$\left(\begin{array}{c} 5000,\\ 10,000 \end{array}\right)$	(10)	(50)	(100)	(0,0)	(1,1)	(0,0)
$x_{6}^{B}$	(0,0)	$\left(\begin{array}{c} 25,000,\\ 50,000 \end{array}\right)$	$\left(\begin{array}{c} 100,\\ 150 \end{array}\right)$	$\left(\begin{array}{c} 200,\\ 300 \end{array}\right)$	$\left(\begin{array}{c} 500,\\ 1000 \end{array}\right)$	(0,0)	(0,0)	(1,1)
$Z^{B}$	***		(0,0)	(0,0)	(0,0)	***	***	***
$Z_{j}^{B}-C_{j}$	***	***	$\left(\begin{array}{c} -1000,\\ -1500 \end{array}\right)$	$\left(\begin{array}{c} -2000,\\ -3000 \end{array}\right)$	$\left(\begin{array}{c} -5000,\\ -10,000 \end{array}\right)$	***	***	***
